# The Synthetic Cannabinoid AKB-48 Induces Cell Death in Murine Cerebellum Through Different Signaling Pathways

**DOI:** 10.3390/ijms27093867

**Published:** 2026-04-27

**Authors:** Fabrizio De Luca, Giorgia Corli, Marta Bassi, Sabrine Bilel, Daniele Merli, Davide Lonati, Azzurra Schicchi, Paola Rossi, Maria Grazia Bottone, Carlo Alessandro Locatelli, Matteo Marti, Elisa Roda

**Affiliations:** 1 Department of Biology and Biotechnology “L. Spallanzani”, University of Pavia, 27100 Pavia, Italy; paola.rossi@unipv.it (P.R.); mariagrazia.bottone@unipv.it (M.G.B.); 2 Department of Translational Medicine, Section of Legal Medicine, LTTA Center and University Center of Gender Medicine, University of Ferrara, 44121 Ferrara, Italy; giorgia.corli@unife.it (G.C.); marta.bassi@unife.it (M.B.); sabrine.bilel@unife.it (S.B.); matteo.marti@unife.it (M.M.); 3 Department of Chemistry, University of Pavia, Viale Taramelli 10, 27100 Pavia, Italy; daniele.merli@unipv.it; 4 INFN Sezione di Milano-Bicocca, Piazza della Scienza 3, 20126 Milano, Italy; 5 Laboratory of Clinical and Experimental Toxicology, and Poison Control Centre and National Toxicology Information Centre, Toxicology Unit, Istituti Clinici Scientifici Maugeri IRCCS, Via Maugeri 10, 27100 Pavia, Italy; davide.lonati@icsmaugeri.it (D.L.); azzurra.schicchi@icsmaugeri.it (A.S.); carlo.locatelli@icsmaugeri.it (C.A.L.); elisa.roda@icsmaugeri.it (E.R.); 6 Collaborative Center for the Italian National Early Warning System, NEWS-D, Department of Anti-Drug Policies and Other Addictions, Presidency of the Council of Ministers, 00186 Rome, Italy

**Keywords:** AKB-48, APINACA, novel psychoactive substances, synthetic cannabinoid, cerebellum, cell death, sex difference

## Abstract

Novel psychoactive substances (NPSs) exhibit extremely strong pharmaco-toxicological activity, often leading to severe adverse effects that pose a serious risk to consumers’ health. Among these, synthetic cannabinoids (SCs) currently represent the majority of drug seizures in Europe. One such compound, AKB-48 (also known as APINACA), was first identified in Japanese herbal smoking blends in 2012. Although it mimics the effects of Δ9-THC, the primary psychoactive component of *Cannabis sativa*, AKB-48 can induce more severe and potentially life-threatening outcomes. Several in vivo studies investigating the acute administration of AKB-48 have reported profound behavioral, neurological, and neurochemical alterations, including disruptions of neurotransmission across multiple brain regions, thus confirming its neurotoxic potential. Given the recognized vulnerability of the cerebellum to NPS, and its critical role in integrating neural circuits affected by psychostimulant drugs, the present study evaluated the toxic effects of repeated AKB-48 exposure on the cerebellar cortex of adult male and female ICR-CD1^®^ mice. Particular attention was paid to the modulation of cell death pathways, alongside assessments of sensorimotor responses. The results demonstrate, for the first time, that repeated AKB-48 administration induces significant morphological, immunohistochemical, and ultrastructural changes in both male and female mice. These alterations included pronounced disruption of cerebellar architecture and marked modulation of cell death pathways, further corroborated by TEM-detected ultrastructural damage and a substantial reduction in the basal visual placing response. Overall, the findings provide clear evidence of AKB-48’s sex-independent neurotoxicity, leading to cerebellar alterations that ultimately result in neuroplasticity impairment.

## 1. Introduction

In last few decades, the international drugs abuse scenario encountered a huge challenge due to newly available unrestricted, psychedelic chemicals, namely novel psychoactive substances (NPSs). NPSs, illegally synthesized altering the chemical structures of traditional drugs of abuse or even molecules abandoned by pharmaceutical drug development, to avoid legal restrictions and control systems [1], have potent pharmaco-toxicological activity producing severe adverse effects, particularly dangerous for consumers’ health [2]. Concerning their detection, routine laboratory tests fail to detect NPS, resulting in a challenge for clinical and forensic toxicology and for the national and international regulatory agencies [3]. In particular, synthetic cannabinoids (SCs), a growing number of human-made mind-altering substances functionally similar to Δ9-THC, currently dominate drug seizures in Europe [4]. SCs can provoke several central and peripheral alterations [2,5,6,7,8,9]. Among SCs, AKB-48, also named APINACA, is an adamantyl-indazole compound with an adamantly moiety linked to the indazole group through a carboxamide bond, originally detected in Japanese herbal smoking blends in 2012 [10,11]. Then, it was described by the US Drug Enforcement Administration (DEA) as one of the most abundant SCs seized [12,13] and categorized in Schedule I of the Controlled Substances Act [14,15,16].

Similarly to other SCs, AKB-48 mirrors the effect of Δ9-THC, the main psychoactive constituent of *Cannabis sativa* plant [9], but it is able to induce worse, life-threatening effects, probably owing to its great affinity for CB1 and CB2 receptors. Consumers reported a rapid onset of the effects, i.e., euphoria, happiness and relaxation, as well as talkativeness, which may last up to 3 h [17], usually paralleled by several reported side effects, such as hypersalivation, excessive sedation, anxiety, increased heart rate, psychomotor impairment, paranoia, and irritability [11,17,18,19].

In line with data reporting intoxication cases in humans, preclinical studies highlighted AKB-48 toxicity, also attempting to explain its action mechanisms. In particular, neurotoxicity has been demonstrated in a recent in vitro study using human bone marrow neuroblastoma, pointing out inflammation and oxidative stress as key mediators involved in the toxicity of AKB-48, with cell death pathway switching from apoptosis to necrosis with increasing doses [20]. Several in vivo studies exploring the effects of acute administration of AKB-48 in rodents reported severe behavioral, neurological and neurochemical alterations, such as alteration of dopaminergic transmission in different brain areas, confirming AKB-48-threatening toxicity and also indicating its potential of abuse [21,22,23]. Further, other in vivo investigation exploring SC-repeated exposure corroborated the above reported findings, revealing neuroplastic alteration in rodent brain, hence supporting the hypothesis of a long-lasting neurotoxicity induced by SCs even characterized by different chemical structure, e.g., AKB-48 and JWH-018 [11,21,24].

Nevertheless, the toxicity of SCs on human health still remains controversial and poorly comprehended in terms of their potential for abuse, dependence and withdrawal, although many case reports showed that acute toxicity may lead to death. A paucity of knowledge exists about the medium- and long-term effects of these drugs, and the understanding of neurotoxic mechanisms is still scarce, but evidence exists that neurotoxicity outcome could induce everlasting brain injuries. Actually, SC-related psychiatric problems and brain damages/abnormalities are significant, as clearly reported by epidemiological investigations and postmortem neuroimaging studies revealing evident damage in specific brain regions [11,25].

Hence, the urgent need emerged for further in-depth studies to broaden knowledge of SC pharmaco-toxicological profile and neurotoxic effects, also assessed after repeated, chronic exposure [26,27]. To this aim, the present in vivo study investigates the potential neurotoxic effects of repeated intraperitoneal (i.p.) injections of AKB-48 (6 mg/kg) in a murine model (adult ICR-CD1^®^ mice) focusing on a specific brain area, i.e., the cerebellum, also monitoring the sensorimotor responses using a behavioral test (i.e., visual placing response test).

Due to acknowledged vulnerability of cerebellum to the effects of NPS, and based on (i) its role in connecting neural circuits affected by psychostimulant drugs, and (ii) its involvement in several cognitive functions and drug abuse, particularly focusing on its important function in associative learning processes that link drug use to contextual cues [28], immunohistochemical evaluations have been performed on brain specimens to assess expression/changes in molecular markers involved in cell death pathways, with the aim of exploring potential neurotoxic events occurring after exposure to AKB-48, which has been shown to significantly affect/modulate a range of cellular mechanisms. In particular, we evaluated the potential histological and ultrastructural changes in cerebellar cortex cytoarchitecture, together with cell death pathways.

This study will give important information about AKB-48 impact on those mechanisms in this CNS area, clarifying the role of cerebellum-mediated outputs in psychostimulant addiction.

## 2. Results

### 2.1. AKB-48 Elicited Dose-Dependent Alterations on Cerebellar Cytoarchitecture and Impacts on Cell Death Pathway

Histochemical and immunohistochemical investigations were performed on sagittal sections of the cerebellar vermis of both male and female vehicle-treated (control) and AKB-48-treated mice after the first, second or third AKB-48 injection.

The weight of mice and the amount of daily consumed food were monitored, but any difference was measured between vehicle- and AKB-48-treated animals, suggesting that the repeated treatment neither affected body weight nor food consumption in male and female mice.

#### 2.1.1. Histochemical Evaluations

**Figure 1:**
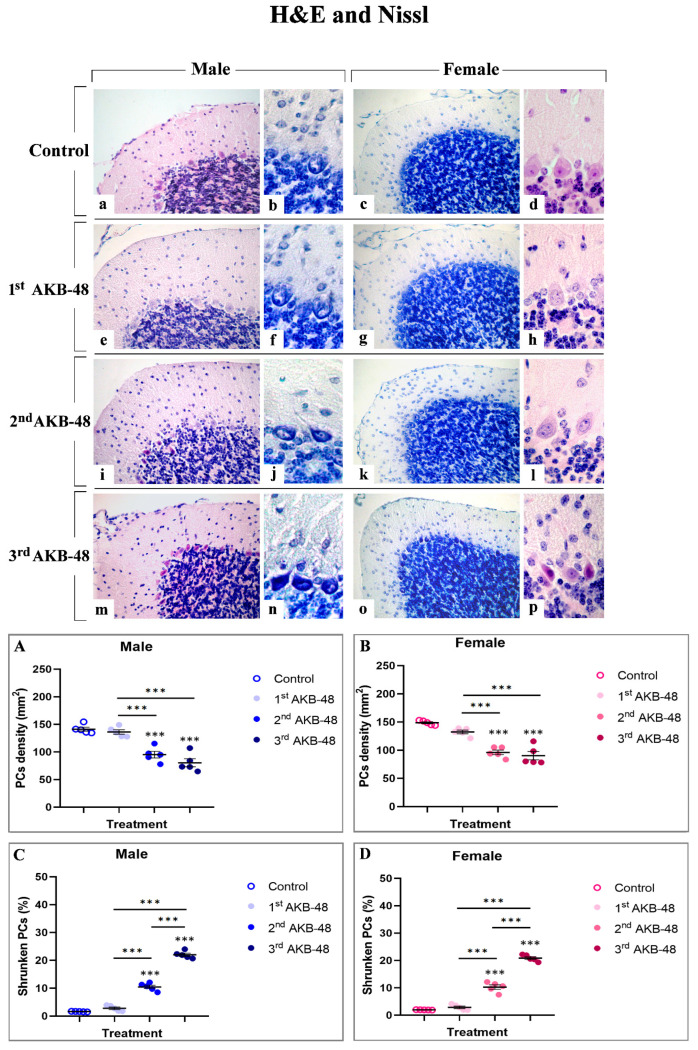
Histological staining with hematoxylin and eosin ((**a**,**e**,**i**,**m**) 40×; (**d**,**h**,**l**,**p**) 100×) and Nissl staining ((**c**,**g**,**k**,**o**) 40×; (**b**,**f**,**j**,**n**) 100×) of the cerebellar cortex from male and female mice, either untreated (control) or treated with increasing doses of AKB-48. Control (**a**–**d**), 1st AKB-48 dose (**e**–**h**), 2nd AKB-48 dose (**i**–**l**), and 3rd AKB-48 dose (**m**–**p**). The histograms show the analysis of PC density (mm^2^) in male and female mice (Panels (**A**) and (**B**), respectively), as well as the percentage of shrunken PCs (Panels (**C**) and (**D**) for males and females, respectively). *** *p* < 0.001. *n* = 5/group.

Morphological picture obtained by H and E (a, d, e, h, I, l, m and p) and Nissl (b, c, f, g, j, k, n and o) staining in control male (a–b) and female (c–d) mice and the first (e–h), second (i–l) or third (m–p) AKB-48-treated animals are illustrated in Figure 1.

Healthy controls displayed a well-preserved physiological cerebellar cytoarchitecture. Conversely, both male- and female-treated mice displayed a dose-dependent alteration in the cerebellar cortex organization, characterized by reduction in Purkinje cell (PCs) density accompanied by a concomitant increase in shrunken cells.

In particular, even though the first AKB-48 administration caused only a slight and non-statistically significant reduction in PCs density, both in males and females compared to their respective controls, a dramatic and highly significant reduction was assessed after the second and third AKB-48 administration. This lessening was determined in both sexes, when compared to their respective control groups and to the first AKB groups. However, any significant difference was detected in PC density between the second and third AKB groups in either sex (Figure 1, Panel A and B, and Tables S1 and S2, for males and females respectively).

A similar pattern emerged when examining the percentage of shrunken PCs. Any significant difference was gauged comparing the first AKB and control group in both sexes. Conversely, as the number of AKB-48 administrations increases, the percentage of shrunken PCs significantly enhanced. After the second and third administration, this percentage rose significantly both in males and females, compared to their respective control and the first AKB group. Notably, after the third administration, a further increase in the number of shrunken cells was assessed compared to the second AKB group in both sexes (Figure 1, Panel C and D, and Tables S3 and S4, for males and females respectively).

#### 2.1.2. Immunohistochemical Investigation

Psychoactive substances, such as synthetic cannabinoids like AKB-48, have been shown to significantly affect a range of cellular mechanisms, including cell death pathway modulation. These NPSs can alter normal cellular homeostasis, triggering specific cell death mechanisms, e.g., apoptosis or necrosis, which are pivotal in maintaining tissue integrity.

**Figure 2:**
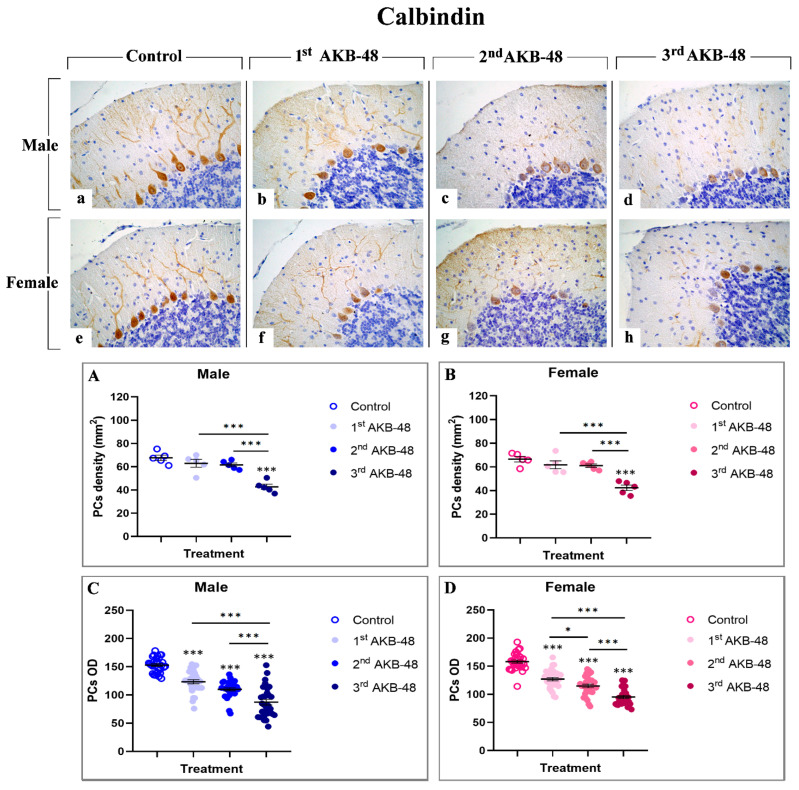
Immunohistochemical staining for calbindin in the cerebellar cortex of male and female mice, either untreated (control) or treated with increasing doses of AKB-48. Control (**a**,**e**), 1st AKB-48 dose (**b**,**f**), 2nd AKB-48 dose (**c**,**g**), and 3rd AKB-48 dose (**d**,**h**). Images (**a**–**h**), 40×. The histograms show the analysis of immunopositive PC density (mm^2^) in male and female mice (Panels (**A**) and (**B**), respectively), as well as immunolabeled PC OD (Panels (**C**) and (**D**) for males and females, respectively). * *p* < 0.05, *** *p* < 0.001. *n* = 5/group.

Calcium-binding proteins, including calbindin, represent a group of cytoplasmic proteins which are able to play a protective role in safeguarding cells against injury [29]. The immunoreaction for calbindin (Figure 2) was localized in the somata and dendritic three of PCs in all evaluated experimental groups in which an intense immunopositivity was evident. Notably, calbindin-immunopositivity was clearly detectable in Purkinje neurons of control male (a) and female (e) mice, while a progressive reduction was observed after repeated AKB-48 administration. This decrease was evident both in terms of immunopositive PC OD and cell density (b and f, c and g, and d and h, for first, second, and third AKB, respectively) in male (b–d) and female (f–h) mice. In particular, while the first and second administration of AKB-48 triggered only a slight reduction in calbindin-immunopositive PC density both in male and female groups compared to controls, an extremely significant lessening was observed after the third dose in both sexes compared to the previous experimental groups (Figure 2, Panel A and B, and Tables S5 and S6, for males and females respectively). Conversely, even after the first AKB-48 administration, a marked reduction in the PC OD was determined in both sexes compared to controls. This OD reduction became even more pronounced after the second AKB-48 administration, albeit with varying degrees of significance between sexes. As already assessed for immunopositive cell density, a further significant OD reduction was measured in the third AKB-treated group, both in males and females, when compared to their respective control, first AKB, and second AKB groups (Figure 2, Panel C and D, and Tables S7 and S8 for males and femalesle respectively).

**Figure 3:**
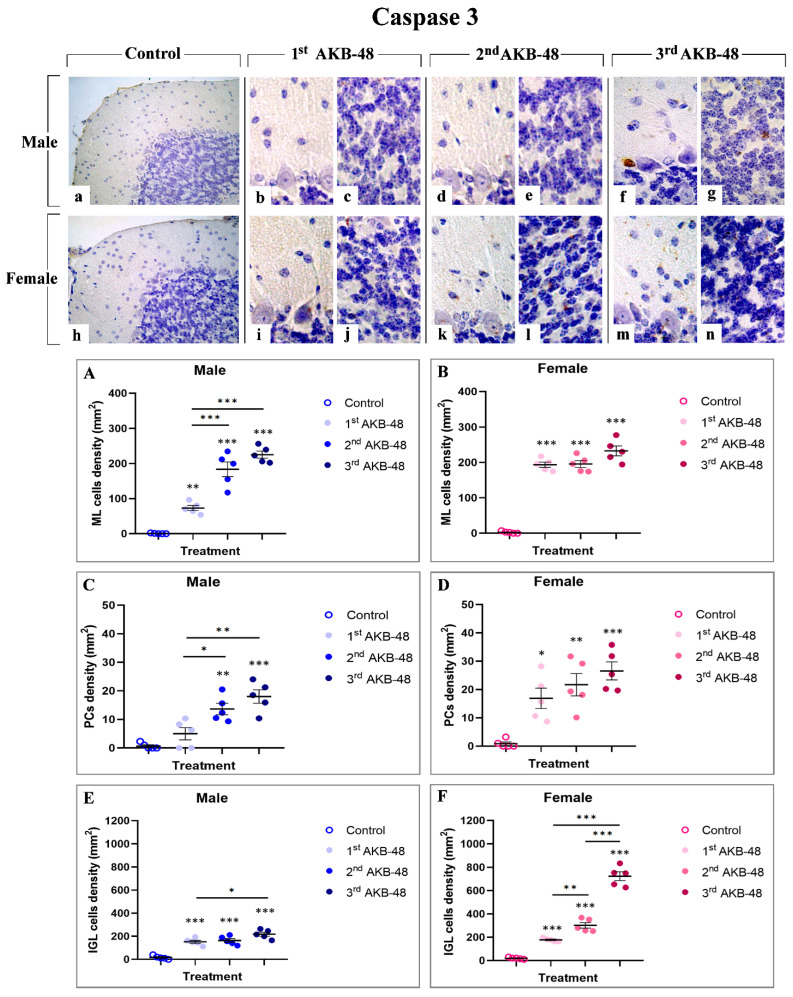
Immunohistochemical staining for caspase 3 in the cerebellar cortex of male and female mice, either untreated (control) or treated with increasing doses of AKB-48. Control (**a**,**h**), 1st AKB-48 dose (**b**,**c**,**i**,**j**), 2nd AKB-48 dose (**d**,**e**,**k**,**l**), and 3rd AKB-48 dose (**f**,**g**,**m**,**n**). Images (**a**,**h**), 40×; (**b**–**g**,**i**–**n**), 100×. The histograms show the analysis of immunopositive ML cell density (mm^2^) in male and female mice (Panels (**A**) and (**B**), respectively), immunolabeled PC density (mm^2^) (Panels (**C**) and (**D**) for males and females, respectively), and immunopositive IGL cell density (mm^2^) in both male and female groups (Panels (**E**) and (**F**), respectively). * *p* < 0.05, ** *p* < 0.01, *** *p* < 0.001. *n* = 5/group.

Caspase 3 is one of the critical effector molecules involved in apoptosis [30]. Immunostaining for caspase 3 (Figure 3) revealed a lack of immunoreactivity in the different layers of the cerebellar cortex, both in control male and female mice (a and h, for males and females, respectively). Nevertheless, even after a single AKB-48 administration, an increase in the density of caspase 3-immunolabeled cells was assessed in the outer molecular layer (ML), the PC layer, and the inner granular layer (IGL) of both sexes. Notably, the density of caspase 3 immunopositive cells continued to rise with increasing AKB-48 administrations (b–c and i–j, d–e and k–l, and f–g and m–n, for the first, second, and third administrations, respectively) in both the male (b–g) and female mouse groups (i–n).

In particular, a significant increase in immunopositive cell density was gauged in the ML of first AKB-48-treated males, compared to controls. A further enhancement was observed in the same experimental group after the second and third administrations, compared to both the first AKB-48-treated and control groups. Differently, an extremely significant increase in caspase 3-immunolabeled cell density was observed in the ML of female mice already after a single AKB-48 injection. This augment was also evident, without any additional significant rise, when comparing the second and third AKB-48-treated groups with the first AKB-48-treated and control female mice (Figure 3, Panel A and B, and Tables S9 and S10, for males and females respectively). Concerning male mice, a slight non-significant increase in immunopositive PC density was detected in first AKB-48-treated animals compared to controls; however, a statistically significant increase was measured after the second and third AKB-48 administrations, compared to both first AKB-48-treated and control mice. Parallelly, in females, all treated groups showed a statistically significant increase in caspase 3-immunolabeled PC density compared to controls. Nonetheless, evidencing a pattern similar to that observed in the ML, any statistically significant difference was assessed among the three AKB-48-treated female groups, i.e., first, second, and third AKB-48 (Figure 3, Panel C and D, and Tables S11 and S12 for males and femalesle respectively). Regarding the IGL, caspase 3-immunolabeled cell density significantly increased in first and second AKB-48-treated males compared to their respective controls, even though no difference was measured between the first and second AKB animals. Differently, a further increase in caspase 3-immunopositive cell density was revealed in the third AKB-treated group, compared to both the first AKB-treated and control males. In females, an extremely significant augment was evident already after a single AKB-48 administration, with a further significant spread observed in the second AKB-treated group. This increase was even more pronounced in the third AKB-treated group, compared to both first and second AKB mice as well as control group of the same sex (Figure 3, Panel E and F, and Tables S13 and S14 for males and femalesmale respectively).

**Figure 4:**
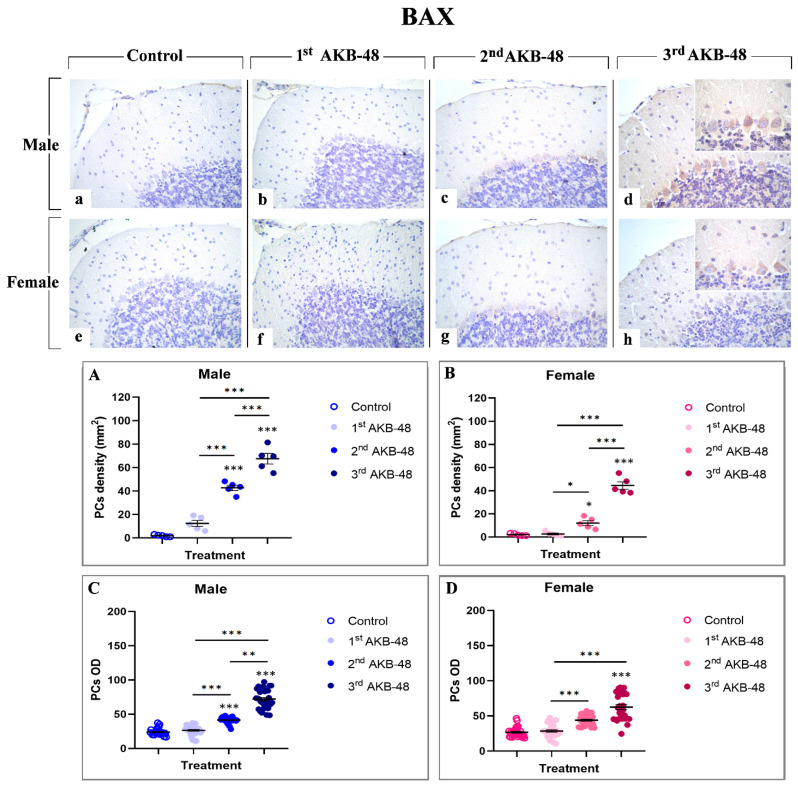
Immunohistochemical staining for BAX in the cerebellar cortex of male and female mice, either untreated (control) or treated with increasing doses of AKB-48. Control (**a**,**e**), 1st AKB-48 dose (**b**,**f**), 2nd AKB-48 dose (**c**,**g**), and 3rd AKB-48 dose (**d**,**h**). Images (**a**–**h**), 40×; insets, 100×. The histograms show the analysis of immunopositive PC density (mm^2^) in male and female mice (Panels (**A**) and (**B**), respectively) as well as immunolabeled PC OD (Panels (**C**) and (**D**) for males and females, respectively). * *p* < 0.05, ** *p* < 0.01, *** *p* < 0.001. *n* = 5/group.

The Bcl-2 protein family includes the pro-apoptotic protein BAX, a key player in the regulation of programmed cell death [31]. Our study revealed an AKB-48-induced increase in BAX expression levels evident in the soma of Purkinje neurons, both in male (b, c, and d, for the first, second, and third administrations, respectively) and female (f, g, and h, for the first, second, and third administrations, respectively) mice. Any immunolabelling was observed in the control group neither in male nor in female animals (a and e, respectively). Notably, while a single AKB-48 administration does not trigger a significant increase in the density of BAX-immunopositive Purkinje neurons in both sexes, after the second administration the density of BAX immunoreactive cells significantly rose both in male and female mice, compared to controls and the first AKB-48 group. An even more pronounced increase was measured in both sexes when comparing the third AKB-48 group to their respective control, first and second AKB-48 groups (Figure 4, Panel A and B, and Tables S15 and S16, for males and females respectively). Concerning the OD assessment, a similar trend was revealed. In fact, any significant difference in BAX-immunopositive Purkinje cell OD was determined when comparing first AKB-48 mice and control groups, regardless of sex. However, similarly to data above described for immunolabeled cell density, a significant increase in BAX-immunopositive cell OD was measured after the second AKB administration, further exacerbated after the third AKB-48 administration (Figure 4, Panel C and D, and Tables S17 and S18, for males and females respectively).

**Figure 5:**
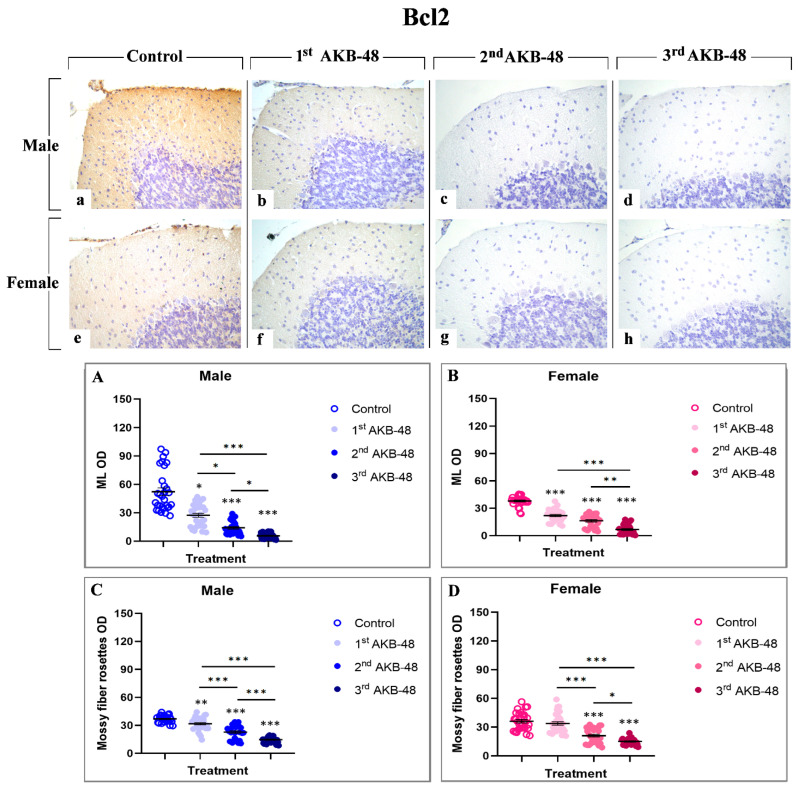
Immunohistochemical staining for Bcl2 in the cerebellar cortex of male and female mice, either untreated (control) or treated with increasing doses of AKB-48. Control (**a**,**e**), 1st AKB-48 dose (**b**,**f**), 2nd AKB-48 dose (**c**,**g**), and 3rd AKB-48 dose (**d**,**h**). Images (**a**–**h**), 40×. The histograms show the analysis of immunopositive ML OD in male and female mice (Panels (**A**) and (**B**), respectively) as well as immunolabeled mossy fiber rosette OD (Panels (**C**) and (**D**) for males and females, respectively). * *p* < 0.05, ** *p* < 0.01, *** *p* < 0.001. *n* = 5/group.

The Bcl-2 protein is a well-established anti-apoptotic factor [32]. The Bcl-2 immunolabeling was localized in the ML and in mossy fiber rosettes, clearly visible in the control group of both male and female mice (a and e, respectively), and gradually decreased in AKB-48-treated animals, in a similar manner both in males (b, c and d, for the first, second and third administrations, respectively) and females (f, g and h, for the first, second and third administrations, respectively). After a single AKB-48 administration, a significant reduction in Bcl-2 immunopositive OD was observed in the ML of both male and female mice, compared to control animals. In the same area, Bcl-2 immunostaining further lessened after the second AKB-48 administration in both sexes, compared to both controls and first AKB-48 group. This decline was significantly more pronounced after the third AKB-48 administrations, both in males and females (Figure 5, Panel A and B, and Tables S19 and S20, for males and females respectively). A similar immunostaining trend was observed in the mossy fiber rosettes, with the anti-apoptotic markers significantly reducing after repeated AKB-48 administration, even showing a different extent comparing males and females. Notably, a highly significant diminution in Bcl-2 immunopositive mossy fiber rosette OD was assessed after the first AKB-48 administration in males only, with the females remaining unaffected. Conversely, after the second AKB-48 administration, a significant lessening of Bcl-2 immunopositive mossy fiber rosette OD was evidenced both in males and females, compared to controls and first AKB-48 group. As for the ML immunolabelling, a further significant decrease in Bcl-2 immunopositive mossy fiber rosette OD was determined after the third AKB-48 administration in both sexes (Figure 5, Panel C and D, and Tables S21 and S22, for males and females respectively).

**Figure 6:**
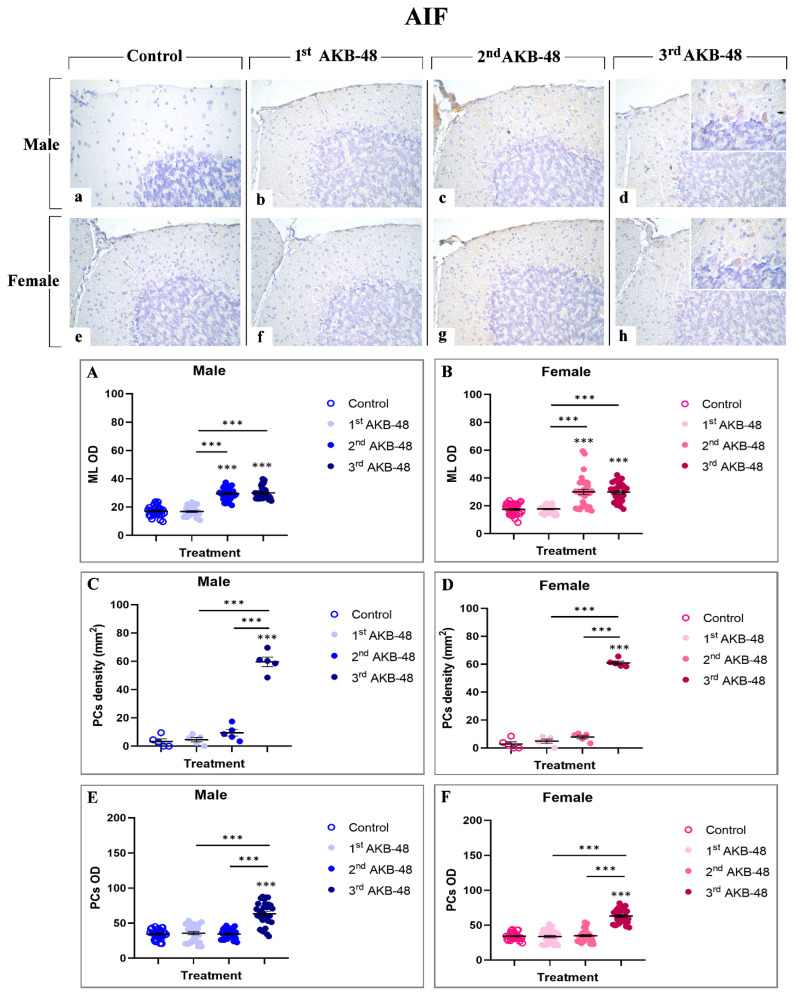
Immunohistochemical staining for AIF in the cerebellar cortex of male and female mice, either untreated (control) or treated with increasing doses of AKB-48. Control (**a**,**e**), 1st AKB-48 dose (**b**,**f**), 2nd AKB-48 dose (**c**,**g**), and 3rd AKB-48 dose (**d**,**h**). Images (**a**–**h**), 40×; insets, 100×. The histograms show the analysis of immunopositive ML OD in male and female mice (Panels (**A**) and (**B**), respectively), immunolabeled PC density (mm^2^) (Panels (**C**) and (**D**) for males and females, respectively), and immunopositive PC OD in both male and female groups (Panels (**E**) and (**F**), respectively). *** *p* < 0.001. *n* = 5/group.

Apoptosis-inducing factor (AIF) is a mitochondrial intermembrane protein involved in caspase-independent cell death mechanisms [33]. Immunohistochemical analysis revealed the presence of this cell death marker in the ML and in the soma of Purkinje cells, even though with different extents, across all experimental groups, in both male (a, b, c and d, for control, first, second, and third AKB-48, respectively) and female (e, f, g, and h, for control, first, second, and third AKB-48, respectively) animals. Notably, concerning the ML, any significant difference in the AIF-immunopositive OD was observed after the first AKB-48 administration, when comparing male and female mice to their respective controls. Conversely, a striking increase in AIF expression was detected in both sexes following the second and third AKB-48 administrations, with significantly higher levels compared to those measured in controls and first AKB-48 group. Interestingly, no differences were revealed when comparing males and females belonging to the second and third AKB-48 groups (Figure 6, Panel A and B, and Tables S23 and S24, for males and females respectively).

Parallelly, the analyses of AIF-immunopositive Purkinje cell density revealed the lack of significant differences either in male or female mice, comparing controls, first and second AKB-48 groups. In contrast, after the third AKB-48 administration, a marked increase in AIF-immunopositive cell density was assessed in both sexes comparing all experimental groups, i.e., control, first and second AKB-48 (Figure 6, Panel C and D, and Tables S25 and S26, for males and females respectively). The same pattern was determined when assessing AIF-immunopositive Purkinje cell OD. Briefly, any significant changes were evidenced comparing the control, first and second AKB-48 groups either in male or female animals. Contrarily, a highly significant rise in AIF-immunopositive Purkinje cell OD values was measured after the third AKB-48 administration (Figure 6, Panel E and F, and Tables S27 and S28 for males and females respectively).

**Figure 7:**
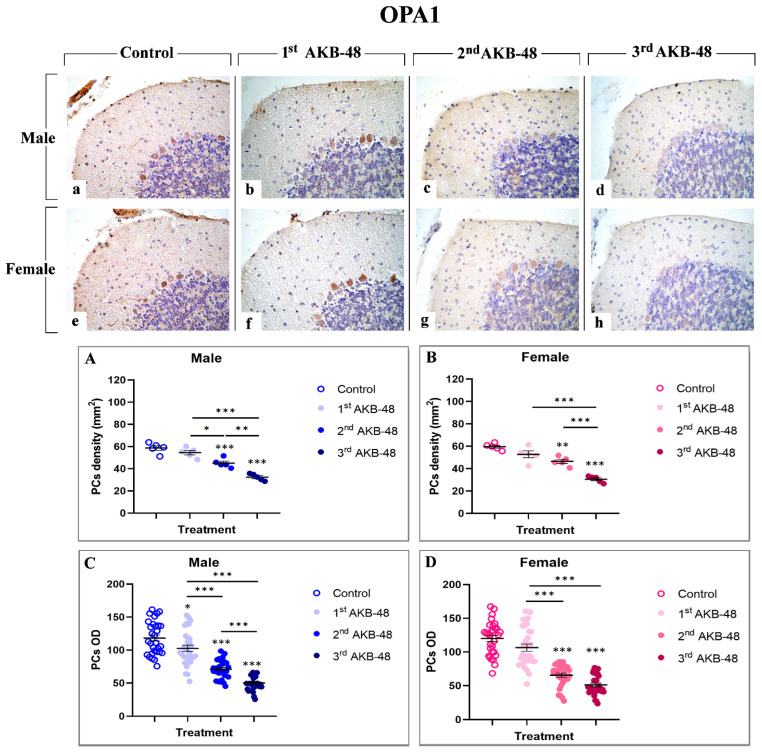
Immunohistochemical staining for BAX in the cerebellar cortex of male and female mice, either untreated (control) or treated with increasing doses of AKB-48. Control (**a**,**e**), 1st AKB-48 dose (**b**,**f**), 2nd AKB-48 dose (**c**,**g**), and 3rd AKB-48 dose (**d**,**h**). Images (**a**–**h**), 40×. The histograms show the analysis of immunopositive PC density (mm^2^) in male and female mice (Panels (**A**) and (**B**), respectively) as well as immunolabeled PC OD (Panels (**C**) and (**D**) for males and females, respectively). * *p* < 0.05, ** *p* < 0.01, *** *p* < 0.001. *n* = 5/group.

Optic Atrophy 1 (OPA1) is a dynamin-like GTPase involved in ferroptosis [34]. In the current investigation, a strong immunolabelling for this cell death marker was observed in the soma of Purkinje cells both in male (a, b, c, and d, for control, first, second, and third AKB-48, respectively) and female (e, f, g, and h, for control, first, second, and third AKB-48, respectively) mice. The analysis of OPA1-immunopositive Purkinje cell density revealed a slight reduction in both sexes after a single AKB-48 administration, compared to controls. Conversely, a significant decrease in the OPA1-immunopositive Purkinje cell density was determined after the second AKB-48 administration, both in male and female mice when compared to controls. The reduction worsened after the third AKB-48 administration compared to all other experimental groups (i.e., controls, first AKB-48 and second AKB-48) (Figure 7, Panel A and B, and Tables S29 and S30, for males and females respectively). Similarly, assessing the OPA1-immunopositive Purkinje cell OD, a slight lessening was detected after the first AKB-48 administration both in male and female animals, compared to their respective controls. Showing a pattern similar to that reported for immunopositive cell density, a significant OPA1-immunopositive cell OD diminution was revealed after the second AKB-48 administration in both sexes, compared to controls. Moreover, the third AKB-48 dose led to a further significant drop in male mice only, while no significant difference was measured comparing the second and third AKB-48 female animals (Figure 7, Panel C and D, and Tables S31 and S32 for males and females respectively).

### 2.2. AKB-48 Induces Ultrastructural Alterations

**Figure 8:**
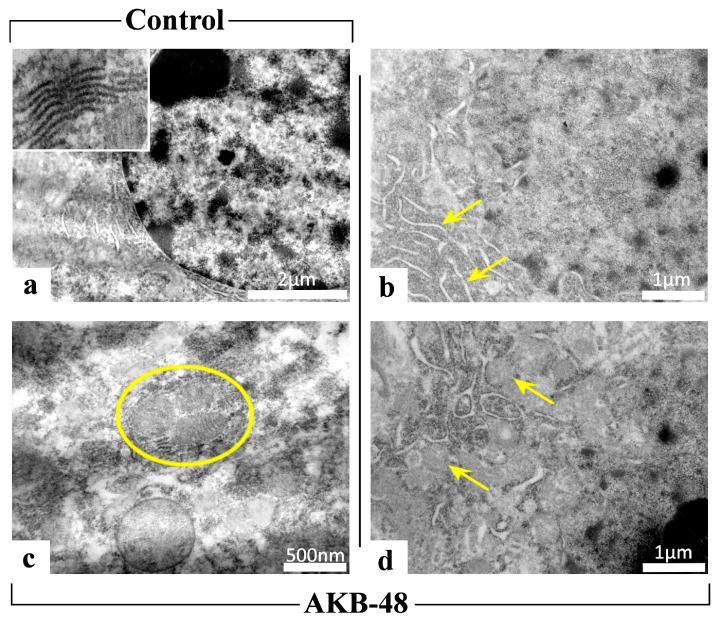
Transmission electron microscopy (TEM) images of control animals ((**a**), 20 K; inset 75 K) and AKB-48-treated mice (AKB-48) ((**b**), 25 K, arrows: ER; (**c**), 50 K, circle: mitophagosome; (**d**), 25 K, arrows: autophagic vesicles). *n* = 3/group.

Transmission electron microscopy images revealed the presence of ultrastructural cellular damage in all experimental groups following AKB-48 treatment (Figure 8). A physiological ultrastructural organization was instead observed in control mice. In all treated mice (i.e., first AKB-48, second AKB-48 and third AKB-48) of both sexes, severe ultrastructural alterations were observed, highlighting the occurrence of cell death events (i.e., autophagy and mitophagy) following administration of the synthetic cannabinoid. In particular, AKB-48 groups displayed: (i) endoplasmic reticulum cisternae with loose and disorganized morphology, (ii) increased number of mitochondria, and (iii) mitophagic events, whereby damaged mitochondria are degraded through autophagic processes leading to the formation of mitophagosomes, a key marker of cellular damage.

### 2.3. Repeated Treatment with AKB-48 Altered the Basal Response to Visual Placing Response Test

**Figure 9:**
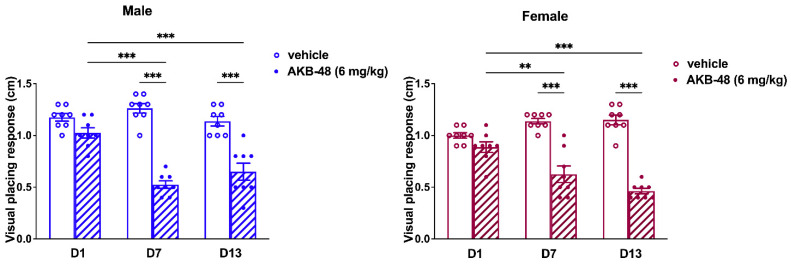
Mean overall effect of the treatment with AKB-48 (6 mg·kg^−1^, i.p.) on visual placing response in male (**left**) and female (**right**) mice, recorded before each injection. Data are expressed as centimeters. Statistical analysis was performed by one-way ANOVA followed by Tukey’s test for multiple comparisons. ** *p* < 0.01, and *** *p* < 0.001; *n* = 8/group.

The visual placing response did not change in vehicle mice over the 13-day observation period. Conversely, AKB-48 (6 mg/kg, i.p.) reduced the visual placing response in both sexes (Figure 9). One-way ANOVA detected a significant effect of AKB-48 treatment on the visual placing reflex in male mice, as measured before the second (D7) and third (D13) injections, compared to the vehicle-treated group (Figure 9; F_(5, 42)_ = 35.6, *p* < 0.001). Accordingly, the visual response observed at D7 and D13 was significantly lower than that observed at D1 (before the first cannabinoid injection). Similar results were observed in the female group. One-way ANOVA detected a significant effect of AKB-48 treatment on the visual placing reflex measured before the second (D7) and third (D13) injections (Figure 9; F_(5, 42)_ = 34.16, *p* < 0.001), highlighting a decrease in visual response compared to the vehicle group and the initial assessment (D1).

**Figure 10:**
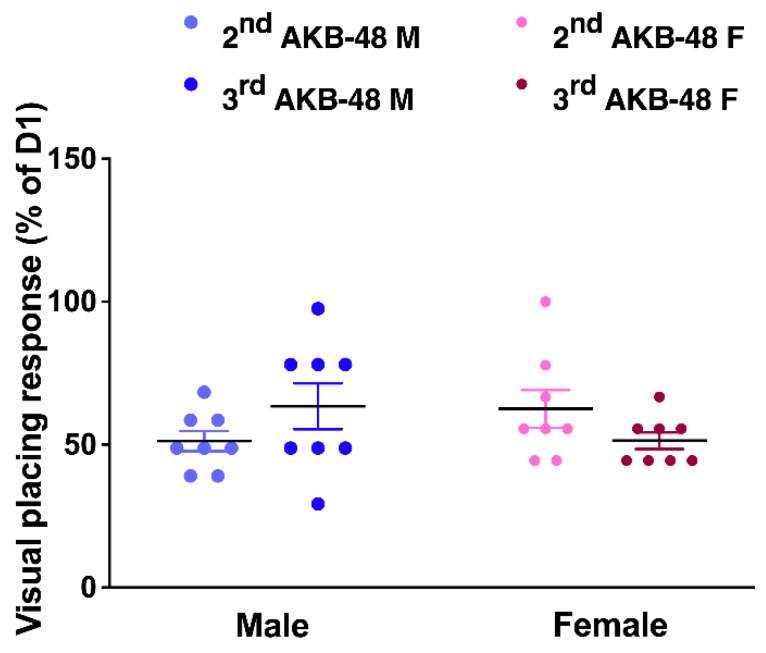
Comparison between the effect induced by the second and third injection of AKB-48 (6 mg·kg^−1^, i.p.) in male (M) and female (F) mice. Data are expressed as a percentage of the values measured before the first injection (D1). Statistical analysis was performed by one-way ANOVA followed by Tukey’s test for multiple comparisons. *n* = 8/group.

The comparison between the altered responses registered after the second and the third injection (expressed as percentage of the values obtained from the test performed before the first injection, at D1) does not reveal differences between sexes (Figure 10; F_(5, 42)_ = 34.16, *p* < 0.001).

## 3. Discussion

Based on the recognized cerebellar vulnerability to NPS effects, also considering cerebellum role in connecting neural circuits affected by psychostimulant drugs, particularly focusing on its important function in associative learning processes linking drug use to contextual cues [28], the current investigation evaluated the potential neurotoxic effects induced by repeated treatment with the synthetic cannabinoid AKB-48 on cerebellar cortex in adult male and female ICR-CD1^®^ mice, exploiting its potential impact on cell death pathways, also monitoring the sensorimotor responses.

The first injection of AKB-48 provokes slight effects in mice, while a progressive increase in cerebellar alterations, including Purkinje neurons lessening/damage as well as changes in cell death key molecules, was revealed at the following injections (second and third). Interestingly, the observed alterations were assessed in both sexes, with females showing more marked effects.

In particular, morphological analyses using H and E and Nissl staining revealed profound alterations in the cerebellar cortex of AKB-48-treated mice. Specifically, as the number of AKB-48 administrations increased, a significant reduction in Purkinje cell density was revealed in both sexes, with a marked lessening occurring after the second administration, paralleled by a concurrent rise in the number of shrunken Purkinje neurons, characterized by severely deformed, rod-shaped condensed nuclei, displaying clear signs of cell degeneration [35,36]. Subsequent immunohistochemical analysis of calbindin expression evidenced a decrease in this Ca^2+^-binding protein levels in the Purkinje neurons of AKB-48-treated mice, in both sexes, with this reduction becoming progressively more pronounced with increasing AKB-48 administrations. This observed pattern supported the hypothesis that specific cell death mechanisms are involved/activated in these neurons. In fact, it is well-known that the progressive calbindin level decline in Purkinje cells undergoing degeneration/death during paraneoplastic cerebellar alterations highlights the critical role of this protein in maintaining intracellular Ca^2+^ homeostasis in cerebellar neurons [29,37]. Furthermore, the apoptotic pathway activation in different cell populations of AKB-48-treated mice was strongly supported by the elevated caspase-3 expression levels measured in all cerebellar cortical layers. This AKB-48-induced increase was observed in both sexes, despite being notably more pronounced in females. Interestingly, specific cortical layers appeared to be more vulnerable than others. In the molecular layer of female mice, for instance, the activation of this programmed cell death mechanism was evident already after a single AKB-48 administration and remained consistently high even after repeated doses. A similar pattern was evidenced in the internal granular layer, with female mice showing a striking threefold increase in caspase-3-immunopositive cells density after the third AKB-48 administration, compared to male animals under the same treatment conditions. As a matter of fact, caspase-8 and caspase-9, key initiator caspases, initiate caspase-3 during the late stages of apoptosis, triggering its proteolytic activity. As a critical member of the cysteine-aspartic protease family, caspase-3 plays a central role in the execution phase of apoptosis, orchestrating the dismantling of cellular components and finalizing the programmed cell death process [38,39,40]. Notably, caspase-3 over-activation may result in excessive apoptosis, with this latter event distinguished in various neurodegenerative disorders, such as Alzheimer’s disease, where the loss of neural cells occurs [41]. Additionally, although a single AKB-48 administration did not seem to significantly affect BAX expression levels, the dose-dependent rise in this pro-apoptotic molecule assesses in Purkinje cells, after repeated AKB administrations in both sexes, further supports the hypothesis that apoptotic pathways or other cell death mechanisms, e.g., necroptosis, are triggered in this specific neuronal population. In fact, this cell death mediator plays a key role in mitochondria-dependent apoptosis and can, in some cases, induce necrotic cell death even without caspase activation [30,42,43,44]. Concerning Bcl-2 immunolabelling, the current results corroborate the assumption that a dose-dependent activation of the apoptotic pathway occurs after AKB-48 treatment, with the onset of similar effects observed both in male and female mice. This anti-apoptotic protein is indeed able to inhibit various apoptotic mechanisms. On the other hand, Bax overexpression can in turn reduce the expression levels of Bcl-2, thereby positively regulating the activation of programmed cell death [32,45,46]. In addition, the data obtained in the present study demonstrate that the apoptotic cell death mechanism in animals treated with increasing doses of AKB-48 may also occur through a caspase-independent pathway, ultimately leading to chromatin condensation and DNA fragmentation [33,46]. In fact, the increase in AIF expression levels measured in the molecular layer and in Purkinje neurons of treated animals, particularly evident after the third AKB-48 injection in both sexes, suggests a potential alternative pathway for the activation of programmed cell death mechanism. Hence, a significant activation of the apoptotic pathway may occur at the highest dose tested, with effects comparable between male and female mice. However, OPA1 data indicate that AKB-48-repeated exposure can trigger non-apoptotic cell death mechanisms, such as ferroptosis, in the Purkinje cells of injected animals. In fact, the progressive decrease in expression levels of this marker, which is critical for maintaining mitochondrial homeostasis and regulating iron-dependent lipid peroxidation leading to ferroptosis, suggests the disruption of key processes such as mitochondrial fusion, structural integrity, and energy metabolism. This disruption results in enhanced mitochondrial ROS production and the inhibition of the integrated stress response, pointing to the possible activation of an alternative mechanism of cell death in these neurons following drug exposure [34,47,48].

The data collected on morphological alterations observed after hematoxylin and eosin and Nissl staining, together with changes in the expression levels of various cell death pathway markers in AKB-48-treated animals, are further corroborated by observations made using transmission electron microscopy. Preliminary TEM data reveal clearly visible ultrastructural alterations indicative of cell death processes (e.g., autophagy and mitophagy), as well as changes in endoplasmic reticulum cisternae in AKB-48-treated animals compared to controls. Nevertheless, it remains necessary to further investigate the effects of different AKB-48 dosing regimens on these alterations, particularly with regard to potential sex differences.

Consistent with the findings from the ex vivo analysis, the in vivo behavioral test demonstrated a substantial decrease in the basal visual placing response (assessed before each injection) in both male and female mice. The visual placing response test evaluates how the mice react when they perceive the impact with the floor, which typically leads to them opening their front paws. This requires the integration of sensory pathways (e.g., visual and tactile) and vestibular functions, which are necessary for correct muscle extension when approaching the ground [49]. Earlier research in the relevant literature has already demonstrated the pivotal role played by the vestibular nucleus (VN), in which CB_1_ receptors targeted by AKB-48 have been located [50], in integrating sensory information relevant to balance and spatial orientation. The VN transmits signals to the vestibulocerebellum (VbC), a crucial component in the perception of self-motion and spatial orientation [51]. Purkinje cells, which are situated in the cerebellar cortex, are responsible for processing this information and transmitting it to the deep cerebellar nuclei of mammals. These nuclei then project to the midbrain and regions such as the VN [52].

Our previous study has pointed out the effects of repeated dosing of AKB-48, highlighting that the alterations observed after each injection were influenced by sex and prolonged treatment [11]. In this context, the current results represent a step forward in understanding the effect of repeated exposure to SCs. In fact, even the basal visual sensorimotor response, measured after six days of washout (before the successive injection), was altered; these data demonstrated that AKB-48 toxicity does not occur only immediately after consumption.

The present study investigated for the first time the morphological, immunohistochemical, and ultrastructural effects of repeated administration of AKB-48 in male and female mice. Clear alterations in the cerebellar architecture were observed in both sexes, together with a marked modulation of the cell death pathway, as evidenced by immunohistochemical analysis of cerebellar tissue sections. These findings were further corroborated by TEM ultrastructural damage, as well as by a substantial decrease in the basal visual placing response assessed before each injection in both male and female mice. Overall, the present data indicate that repeated exposure to AKB-48 leads to profound cerebellar alterations and functional impairment, highlighting a strong sex-independent long-lasting neurotoxicity, finally resulting in neuroplastic alteration. Currently, no specific antidote is available for synthetic cannabinoid poisoning. Patient management mainly relies on supportive care, including fluid and electrolyte correction, while diagnosis remains challenging due to overlapping toxidromes requiring careful neurological and cardiological evaluation [26]. Pharmacological treatment of severe hypertension may include nitrates, benzodiazepines, α-adrenergic antagonists (e.g., prazosin, phentolamine), and β-blockers such as labetalol, although the latter should be used cautiously due to the risk of paradoxical hypertension. Atypical antipsychotics (e.g., haloperidol, ziprasidone, quetiapine, and olanzapine) may be more effective than benzodiazepines as first-line agents, despite potential risks of worsening neurological and cardiac toxicity [53,54]. Emerging therapeutic approaches include the use of naltrexone, nabilone, and naloxone for SC withdrawal [14,15], as well as CB1 receptor antagonists or inverse agonists, which may counteract cannabimimetic effects and could represent potential antidotal strategies [55,56].

A translational perspective has been taken into account when selecting the dosage of AKB48 that mimics a dose typically contained in single packages of synthetic cannabinoid-based products (e.g., “Spice”) [6,7]. This allowed us to provide valuable preliminary information contributing to filling the gap in the knowledge about the consequence of the repeated exposure to synthetic cannabinoids. From a translational point of view, the current findings could be of particular warning for all consumers underestimating AKB-48 toxicity, who would be aware that the neurotoxic effects might occur even long time after consumption. Taken together, despite the many limitations that reside in the translation from animal models to humans, these findings suggest that repeated exposure to SCs may affect psychomotor performances which are related to driving or hazardeous working abilities and may pose a higher risk for road and work accident [57]. Thus, this study provides preclinical information that could contribute to deepening the insight in the risk related to the spread of these drugs with relevant policy and regulatory implications. However, the use of a single dose to evaluate the consequences of repeated exposure to the drug on cerebellar neurotransmission could raise questions. Further studies on animals, testing different doses or structurally different SCs, will be carried out to strengthen these hypotheses.

## 4. Materials and Methods

### 4.1. Animals

Adult ICR (CD-1^®^) male and female mice (weight ranging between 30 and 35 g) were obtained from the Centralized Preclinical Research Laboratory at the University of Ferrara, Italy. Animals were housed (5 per cage; floor area: 80 cm^2^ per mouse; minimum cage height: 12 cm) under constant conditions of temperature (20–22 °C), humidity (45–55%), and photocycle (12 h light/12 h dark) with unlimited access to a commercial diet (4RF25 Mucedola Settimo Milanese, Milan, Italy). All experimental procedures were in accordance with the guidelines laid out by the U.K. Animals (Scientific Procedures) Act of 1986, also in compliance with the European Council Directive 2010/63/EU, and were approved by the Ethics Committee of the University of Ferrara and by Italian Ministry of Health (license n. 223/2021-PR, CBCC2.46.EXT.21). According to ARRIVE guidelines, mice have been treated humanely, with due consideration for reduction in pain and distress, also limiting the number of animals used in the experiments.

### 4.2. Drug Preparation and Dose Selection

N-(1-adamantyl)-1-pentyl-1H-indazole-3-carboxamide (AKB-48) was purchased from LGC Standards S.r.l (Milan, Italy), dissolved in 5% ethanol (for molecular biology, ≥99.8%; BioUltra; Sigma-Aldrich, Italy) + 2%TWEEN^®^80 (Sigma-Aldrich, Milan, Italy) + 96% saline (0.9% NaCl; Eurospital, Trieste, Italy), and injected intraperitoneally (i.p.) at a dose of 6 mg·kg^−1^ in a volume of 4 μL·g^−1^. The same ethanol solution, saline, and TWEEN^®^80 was used as the vehicle. The chosen dose was selected based on our previous studies [11,21,22,23]. In particular, our previous study assessed the acute in vitro and in vivo activity of AKB-48 (0.01–6 mg·kg^−1^, i.p.), providing a comparison with that of delta-9-THC and JWH-018 (a first generation synthetic cannabinoid considered as a golden standard for the study of new compounds emerging every year in the illicit market) [22]. Moreover, the pharmaco-toxicological effects, pharmacokinetics, and neuroplasticity at cannabinoid CB_1_ receptors have been investigated focusing on a high dosage [11]. From a translational perspective, the injected dose of 6 mg·kg^−1^ employed in mice mimics a dose of approximately 29.16 mg in humans (Human Equivalent Dose, or HED of 0.486 mg·kg^−1^ for a 60 kg male [58]). As described by several literature reports, a single package of “Spice” commonly contained an SC amount equivalent to 32.1 and 57 mg·g^−1^ [59,60]. Specifically, 76 mg·g^−1^ of a AKB-48 halogenated derivative has been detected in herbal mixtures seized on European soil [60]. Moreover, it has been shown rapid tolerance mechanisms may lead SCs’ consumers to increase intake up to 30 mg·day^−1^ [61,62,63,64]. The results obtained in our previous study have provided foundation to a more specific study aimed at evaluating the toxic effects of repeated AKB-48 exposure on the cerebellar cortex of adult mice considering morphological, immunohistochemical, ultrastructural and behavioral assays. In line with our primary objective, we have considered the same dose and treatment regimen applied in our previous study [11].

**Figure 11:**
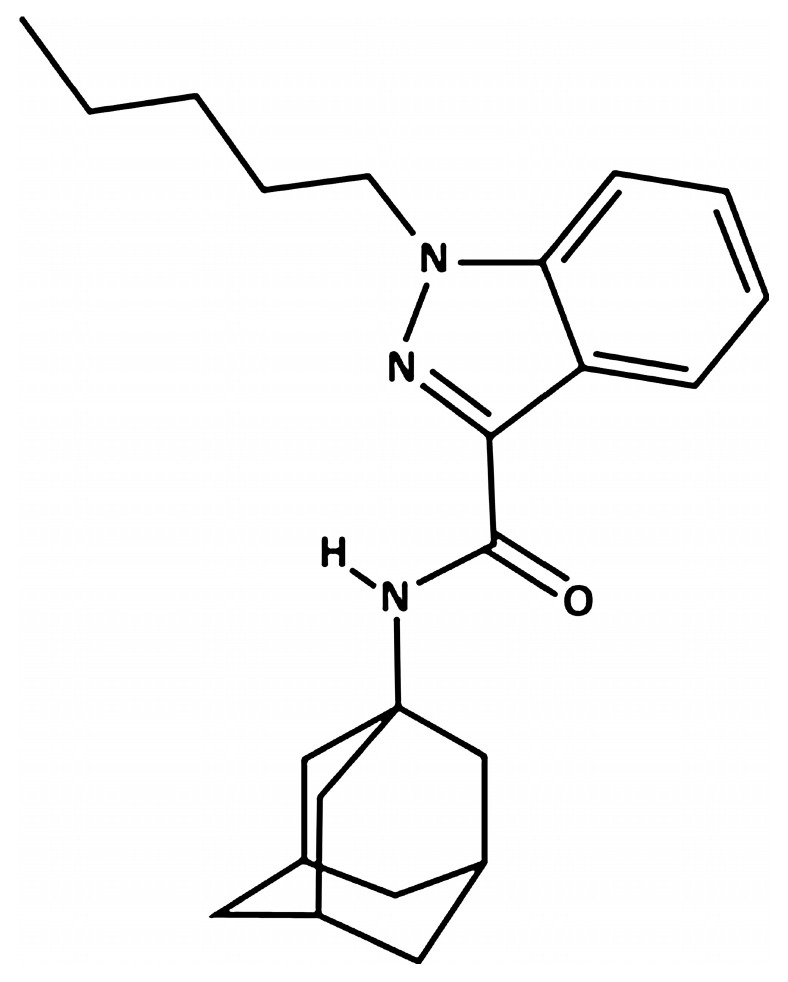
Chemical structure of AKB-48 (APINACA).

Chemical structure of AKB-48 and its corresponding Ki values for the CB1 and CB2 receptors in both human and mouse are reported in Figure 11 and Table 1, respectively.

### 4.3. Animal Treatment

**Figure 12:**
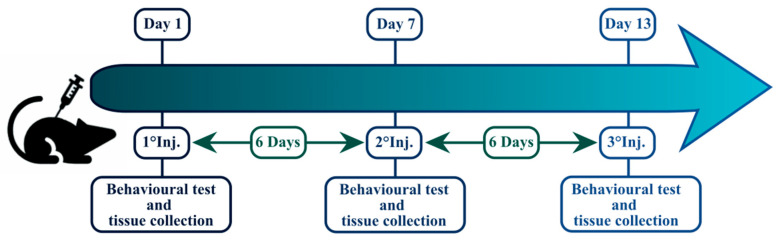
Experimental design and timeline.

Mice were injected with AKB-48 (6 mg·kg^−1^, i.p.) or its vehicle (0.004 mL·g^−1^, i.p.) once per week for three succeeding weeks (13 days), for a total of three i.p. injections (D1, D7, and D13), maintaining a 6-day period of washout between each injection. Forty animals (20 females, 20 males) were randomly selected after the first (D1), second (D7), or third (D13) AKB-48/vehicle injection to execute histological and immunohistochemical studies (Figure 12). Separate sets of mice were treated and assigned to behavioral experiments or blood analyses [11].

### 4.4. Cerebellar Tissue Sampling

Mice were euthanized by cervical dislocation and brains were immediately excised as recently described [30,65,66], washed in 0.9% NaCl and fixed by immersion for 2 days at room temperature (RT) in 4% paraformaldehyde in 0.1-M phosphate buffer (pH 7.4). Tissues were then maintained in absolute ethanol for 1 h, followed by acetone and lastly embedded in Paraplast X-TRA (Sigma Aldrich, Milan, Italy). Serial sectioning of the cerebellar vermis was done using a manual rotary microtome. Sections 8-microns thick were cut in the sagittal plane and collected on silane-coated slides.

### 4.5. Histopathology and Immunohistochemistry

Histochemical staining methods, hematoxylin and eosin (H and E) and Nissl, were used to achieve an overview of tissue morphology [67,68,69,70,71]. Subsequently, using the brightfield examination of H and E and Nissl-stained samples at low magnification, neuroanatomical site identification was achieved, allowing the selection of exact sections of cerebellum, which were subsequently processed for histology and immunohistochemical analysis to gauge expected neurotoxicity [72]. Five slides (about twenty randomized sections) per animal were analyzed, for a total of *n* = 5 animals per experimental condition.

Histochemical and immunohistochemical reactions were done at the same time on slides from different experimental conditions to prevent possible staining differences due to slight changes in the procedures. Immunohistochemistry was performed using commercial antibodies on murine cerebellar specimens to investigate the expression and distribution of specific cell death markers (Table 2).

Paraffin-embedded sections were deparaffinized by using xylene, subsequently rehydrated through a series of graded alcohol treatments and finally rinsed in phosphate-buffered saline (PBS, Sigma, Milan, Italy). Then, sections were incubated overnight at RT in a dark moist chamber with the primary antibody (Table 2). A biotinylated secondary antibody (Table 3) and an avidin biotinylated horseradish peroxidase complex (Vector Laboratories, Burlingame, CA, USA) were used to uncover the sites of antibody/antigen interaction. The 3,3′-diaminobenzidine tetrahydrochloride peroxidase substrate (Sigma, St. Louis, MO, USA) was employed as the chromogen.

The nuclear counterstaining was done by employing Carazzi’s hematoxylin. Subsequently, sections were dehydrated by using ethanol, cleared in xylene and mounted in Eukitt (Kindler, Freiburg, Germany). As negative controls, some sections were incubated only in PBS, in the absence of the primary antibodies; no immunopositivity was detected in this experimental condition.

### 4.6. Histochemical and Immunohistochemical Analysis

Sections were analyzed in brightfield microscopy with an Olympus BX51 optical microscope (BX51TF), and images were acquired using an Olympus CAMEDIA C4040ZOOM camera (Olympus Italia S.r.l., Segrate, MI, Italy). For the assessed marker, 5 slides (about twenty randomized sections) per animal were examined, for a total of *n* = five animals per experimental condition. In all experimental groups, cerebellar specimens with diverse immunolabeling extent were considered. The extent of immunohistochemical labeling was evaluated on acquired digitized section images under exposure time preventing any possible pixel saturation effect. Labeling intensity was subsequently measured using densitometric analysis (Image-J 1.48i; NIH, Bethesda, MA, USA), as previously reported [30,73,74,75]. The immunohistochemical intensity (reported as OD) was evaluated in three randomized images per section (making at least 10 measurements/image) per five slides per animal, for a total of *n* = 5 animals from each experimental condition, with the operator blinded to the experimental group. The labelling was calculated as the mean intensity value over the area for all sections. Moreover, immunoreactive cells density (number of immunopositive cells/area in mm^2^) was assessed as previously described [75].

### 4.7. Transmission Electron Microscopy Evaluations

Treated and control cerebellar samples were processed for TEM analysis as previously described [69,76]. Briefly, cerebellar tissue (small sample of cerebellar vermis, about 1 mm^3^) was fixed by immersion in 1.5% glutaraldehyde for 2 h (Polysciences, Inc., Warrington, PA, USA) buffered with 0.07 M cacodylate buffer (pH 7.4), containing 7% sucrose, and followed by post-fixation for 2 h in OsO_4_ (Sigma Chemical Co., St. Louis, MO, USA) in 0.1 M cacodylate buffer (pH 7.4) at 4 °C, dehydrated in acetone and lastly embedded using epoxy resin. Subsequently, ultrathin sections (70–80 nm) were cut on a Reichert OM-U3 ultramicrotome, collected on nickel grids, and then stained with lead citrate. Lastly, samples were observed under a JEM 1200 EX II (JEOL, Peabody, MA, USA) electron microscope, equipped with a MegaView G2 CCD camera (Olympus OSIS, Tokyo, Japan) and operating at 100 kV and then analyzed using the iTEM software (version n. v.5.1). Three slides (about 15 randomized fields) per animal were analyzed, for a total of *n* = 5 animals per experimental condition.

### 4.8. Behavioral Test

The effect of AKB-48 on sensorimotor responses has been evaluated using a behavioral test (*Visual placing response test*) routinely adopted in our laboratory for the assessment of synthetic cannabinoid effects in mice [11,22,77,78,79]. Experiments were carried out between 8:30 AM and 2:00 PM in a thermostatic (humidity: 45–55%, temperature: 20–22 °C) and light (150 lx) controlled room with a background noise of 40 ± 4 dB, by trained observers working in blind and in pairs. Animals’ behavior was videotaped and analyzed offline by a different trained operator.

The *Visual placing response test* was done using a tail suspension-modified apparatus able to bring the animal towards the floor at a constant speed of 10 cm/s. The downward movement of the animal was videotaped, and the frame-by-frame video was studied to evaluate the beginning of the animal’s reaction while it was near the floor. When the mouse started the reaction, an electronic ruler evaluated the perpendicular distance in mm between its eyes and the floor. A naïve mouse typically perceives the floor and prepares for contact at a distance of about 1.5 ± 0.25 cm [11]. Evaluation of the visual placing response was measured before the first (D1), second (D7), and third (D13) injection to estimate the basal visual placing reflex of the mouse. Behavioral analyses were conducted using 8 animals per experimental group.

### 4.9. Statistical Analysis

Histochemical and immunohistochemical data were reported as mean ± SEM. For data that passed the D’Agostino and Pearson, Anderson–Darling, Shapiro–Wilk, or Kolmogorov–Smirnov tests, the analysis was performed using one-way ANOVA followed by Bonferroni’s post hoc test for multiple comparisons. Otherwise, for non-normally distributed results, the analysis was made employing the Kruskal–Wallis test followed by Dunn’s test. In sensorimotor response experiments, data are expressed in centimeters and percentages of values determined before the first injection (D1). All behavioral data are reported as mean ± SEM and investigated by repeated measures analysis of variance (ANOVA). The total average effect induced by treatments was studied by one-way ANOVA followed by Tukey’s test for multiple comparisons. The differences were considered statistically significant for *p* < 0.05. All statistical analyses were performed using GraphPad Prism 8.0 (GraphPad Software Inc., San Diego, CA, USA).

## Figures and Tables

**Table 1:** Binding parameters of AKB48 to mouse and human CB1 and CB2 receptors. Data are expressed as mean ± SEM [22].

*Compound*	*Mouse CB1* *Ki* (nM)	*Mouse CB2* *Ki* (nM)	*Human CB1* *Ki* (nM)	*Human CB2* *Ki* (nM)
**AKB-48**	5.34 ± 0.44	1.93 ± 0.14	3.24 ± 0.28	1.68 ± 0.12

**Table 2:** Primary antibodies and respective dilutions employed for immunohistochemical experimental procedures.

	Antigen	Immunogen	Manufacturer, Species, Mono-Polyclonal, cat./lot	Dilution
**Primary** **antibodies**	Calbindin D28K	Purified antibody raised against *E. coli*-derived recombinant human Calbindin D Met1-Asn261	Invitrogen (Waltham, MA, USA), Rabbit polyclonal IgG, Cat# PA1-16991	1:50
	Anti-caspase-3 (31A1067)	Purified antibody raised against amino acids 50–86 of caspase-3 (human origin)	Santa Cruz Biotechnology (Santa Cruz, CA, USA), Mouse monoclonal IgG, Cat# sc-56053	1:100
	Anti-apoptosis-inducing factor (E-1)	Purified antibody raised against amino acids 1–300 of AIF (human origin)	Santa Cruz Biotechnology (Santa Cruz, CA, USA), Mouse monoclonal IgG, Cat# sc-13116	1:100
	Anti-Bcl-2-associated X protein (P-19)	Purified antibody raised against a peptide mapping at the amino terminus of Bax (mouse origin)	Santa Cruz Biotechnology (Santa Cruz, CA, USA), Rabbit polyclonal IgG, Cat# sc-526	1:100
	Anti-B-Cell Leukemia/Lymphoma 2 protein (N-19)	Purified antibody raised against a peptide mapping at the N-terminus of Bcl-2 (human origin)	Santa Cruz Biotechnology (Santa Cruz, CA, USA), Rabbit polyclonal IgG, Cat# sc-492	1:100
	Anti-OPA1	Purified antibody raised against a synthetic peptide made to an internal region within residues 500–600 of human OPA1.	Invitrogen (Waltham, MA, USA), Rabbit polyclonal IgG, Cat# PA1-16991	1:400

**Table 3:** Secondary antibodies and respective dilutions employed for immunohistochemical experimental procedures.

	Antigen	Immunogen	Manufacturer, Species, Mono-Polyclonal, cat./lot	Dilution
**Secondary** **antibodies**	Biotinylated goat anti-rabbit IgG	Gamma immunoglobulin	Vector Laboratories (Burlingame, CA, USA), Goat, Cat# PK-6101	1:200
	Biotinylated horse anti-mouse IgG	Gamma immunoglobulin	Vector Laboratories (Burlingame, CA, USA), Horse, Cat# PK-6102	1:200

## Data Availability

Data are provided within the manuscript or Supplementary Information Files.

## Supplementary Materials

The following supporting information can be downloaded at https://www.mdpi.com/article/10.3390/ijms27093867/s1.
