# Supplementary material for: The Synthetic Cannabinoid AKB-48 Induces Cell Death in Murine Cerebellum Through Different Signaling Pathways
Source: Int J Mol Sci. 2026 Apr 27;27(9):3867. doi: 10.3390/ijms27093867 (PMC13163767; doi:10.3390/ijms27093867)
Supplement: Supplementary file 1 [file ijms-27-03867-s001.zip › ijms-4247838-supplementary.pdf]

Table S1. Statistical analysis for the quantitative valuation of PCs density (mm^2) in male; ns: not significant, ***p<0.001.

Experimental group		Experimental group	*p*-value
Control (141.39 ± 3.56)	*vs*	1st AKB-48 (136.35 ± 4.04)	ns
Control (141.39 ± 3.56)	*vs*	2nd AKB-48 (95.31 ± 6.01)	***
Control (141.39 ± 3.56)	*vs*	3rd AKB-48 (80.53 ± 7.33)	***
1st AKB-48 (136.35 ± 4.04)	*vs*	2nd AKB-48 (95.31 ± 6.01)	***
1st AKB-48 (136.35 ± 4.04)	*vs*	3rd AKB-48 (80.53 ± 7.33)	***
2nd AKB-48 (95.31 ± 6.01)	*vs*	3rd AKB-48 (80.53 ± 7.33)	ns

Table S2. Statistical analysis for the quantitative valuation of PCs density (mm^2) in female; ns: not significant, ***p<0.001.

Experimental group		Experimental group	*p*-value
Control (148.78 ± 1.77)	*vs*	1st AKB-48 (132.49 ± 3.15)	ns
Control (148.78 ± 1.77)	*vs*	2nd AKB-48 (96.23 ± 4.10)	***
Control (148.78 ± 1.77)	*vs*	3rd AKB-48 (90.44 ± 7.39)	***
1st AKB-48 (132.49 ± 3.15)	*vs*	2nd AKB-48 (96.23 ± 4.10)	***
1st AKB-48 (132.49 ± 3.15)	*vs*	3rd AKB-48 (90.44 ± 7.39)	***
2nd AKB-48 (96.23 ± 4.10)	*vs*	3rd AKB-48 (90.44 ± 7.39)	ns

Table S3. Statistical analysis for the quantitative valuation of shrunken PCs (%) in male; ns: not significant, ***p<0.001.

Experimental group		Experimental group	*p*-value
Control (1.63 ± 0.03)	*vs*	1st AKB-48 (2.82 ± 0.44)	ns
Control (1.63 ± 0.03)	*vs*	2nd AKB-48 (10.50 ± 0.62)	***
Control (1.63 ± 0.03)	*vs*	3rd AKB-48 (22.03 ± 0.57)	***
1st AKB-48 (2.82 ± 0.44)	*vs*	2nd AKB-48 (10.50 ± 0.62)	***
1st AKB-48 (2.82 ± 0.44)	*vs*	3rd AKB-48 (22.03 ± 0.57)	***
2nd AKB-48 (10.50 ± 0.62)	*vs*	3rd AKB-48 (22.03 ± 0.57)	***

Table S4. Statistical analysis for the quantitative valuation of shrunken PCs (%) in female; ns: not significant, ***p<0.001.

Experimental group		Experimental group	*p*-value
Control (1.98 ± 0.04)	*vs*	1st AKB-48 (2.90 ± 0.45)	ns
Control (1.98 ± 0.04)	*vs*	2nd AKB-48 (10.29 ± 0.81)	***
Control (1.98 ± 0.04)	*vs*	3rd AKB-48 (20.91 ± 0.53)	***
1st AKB-48 (2.90 ± 0.45)	*vs*	2nd AKB-48 (10.29 ± 0.81)	***
1st AKB-48 (2.90 ± 0.45)	*vs*	3rd AKB-48 (20.91 ± 0.53)	***
2nd AKB-48 (10.29 ± 0.81)	*vs*	3rd AKB-48 (20.91 ± 0.53)	***

Table S5. Statistical analysis for the quantitative valuation of calbindin-immunopositive PCs cell density (mm^2) in male; ns: not significant, ***p<0.001.

Experimental group		Experimental group	*p*-value
Control (67.76 ± 2.33)	*vs*	1st AKB-48 (62.99 ± 3.37)	ns
Control (67.76 ± 2.33)	*vs*	2nd AKB-48 (61.66 ± 1.63)	ns
Control (67.76 ± 2.33)	*vs*	3rd AKB-48 (42.77 ± 2.24)	***
1st AKB-48 (62.99 ± 3.37)	*vs*	2nd AKB-48 (61.66 ± 1.63)	ns
1st AKB-48 (62.99 ± 3.37)	*vs*	3rd AKB-48 (42.77 ± 2.24)	***
2nd AKB-48 (61.66 ± 1.63)	*vs*	3rd AKB-48 (42.77 ± 2.24)	***

Table S6. Statistical analysis for the quantitative valuation of calbindin-immunopositive PCs cell density (mm^2) in female; ns: not significant, ***p<0.001.

Experimental group		Experimental group	*p*-value
Control (66.62 ± 2.32)	*vs*	1st AKB-48 (61.83 ± 3.30)	ns
Control (66.62 ± 2.32)	*vs*	2nd AKB-48 (61.11 ± 1.44)	ns
Control (66.62 ± 2.32)	*vs*	3rd AKB-48 (42.40 ± 2.38)	***
1st AKB-48 (61.83 ± 3.30)	*vs*	2nd AKB-48 (61.11 ± 1.44)	ns
1st AKB-48 (61.83 ± 3.30)	*vs*	3rd AKB-48 (42.40 ± 2.38)	***
2nd AKB-48 (61.11 ± 1.44)	*vs*	3rd AKB-48 (42.40 ± 2.38)	***

Table S7. Statistical analysis for the quantitative valuation of calbindin-immunopositive PCs OD in male; ns: not significant, ***p<0.001.

Experimental group		Experimental group	*p*-value
Control (153.11 ± 2.42)	*vs*	1st AKB-48 (123.50 ± 3.52)	***
Control (153.11 ± 2.42)	*vs*	2nd AKB-48 (110.10 ± 2.74)	***
Control (153.11 ± 2.42)	*vs*	3rd AKB-48 (87.33 ± 4.91)	***
1st AKB-48 (123.50 ± 3.52)	*vs*	2nd AKB-48 (110.10 ± 2.74)	ns
1st AKB-48 (123.50 ± 3.52)	*vs*	3rd AKB-48 (87.33 ± 4.91)	***
2nd AKB-48 (110.10 ± 2.74)	*vs*	3rd AKB-48 (87.33 ± 4.91)	***

Table S8. Statistical analysis for the quantitative valuation of calbindin-immunopositive PCs OD in female; *p<0.05, ***p<0.001.

Experimental group		Experimental group	*p*-value
Control (158.20 ± 2.77)	*vs*	1st AKB-48 (127.12 ± 3.05)	***
Control (158.20 ± 2.77)	*vs*	2nd AKB-48 (114.92 ± 3.17)	***
Control (158.20 ± 2.77)	*vs*	3rd AKB-48 (95.25 ± 2.70)	***
1st AKB-48 (127.12 ± 3.05)	*vs*	2nd AKB-48 (114.92 ± 3.17)	*
1st AKB-48 (127.12 ± 3.05)	*vs*	3rd AKB-48 (95.25 ± 2.70)	***
2nd AKB-48 (114.92 ± 3.17)	*vs*	3rd AKB-48 (95.25 ± 2.70)	***

Table S9. Statistical analysis for the quantitative valuation of Caspase 3-immunopositive ML cell density (mm^2) in male; ns: not significant, **p<0.01, ***p<0.001.

Experimental group		Experimental group	*p*-value
Control (0.68 ± 0.47)	*vs*	1st AKB-48 (73.28 ± 7.31)	**
Control (0.68 ± 0.47)	*vs*	2nd AKB-48 (183.98 ± 21.00)	***
Control (0.68 ± 0.47)	*vs*	3rd AKB-48 (225.56 ± 10.17)	***
1st AKB-48 (73.28 ± 7.31)	*vs*	2nd AKB-48 (183.98 ± 21.00)	***
1st AKB-48 (73.28 ± 7.31)	*vs*	3rd AKB-48 (225.56 ± 10.17)	***
2nd AKB-48 (183.98 ± 21.00)	*vs*	3rd AKB-48 (225.56 ± 10.17)	ns

Table S10. Statistical analysis for the quantitative valuation of Caspase 3-immunopositive ML cell density (mm^2) in female; ns: not significant, ***p<0.001.

Experimental group		Experimental group	*p*-value
Control (2.61 ± 1.30)	*vs*	1st AKB-48 (193.78 ± 7.55)	***
Control (2.61 ± 1.30)	*vs*	2nd AKB-48 (195.72 ± 9.72)	***
Control (2.61 ± 1.30)	*vs*	3rd AKB-48 (232.92 ± 14.04)	***
1st AKB-48 (193.78 ± 7.55)	*vs*	2nd AKB-48 (195.72 ± 9.72)	ns
1st AKB-48 (193.78 ± 7.55)	*vs*	3rd AKB-48 (232.92 ± 14.04)	ns
2nd AKB-48 (195.72 ± 9.72)	*vs*	3rd AKB-48 (232.92 ± 14.04)	ns

Table S11. Statistical analysis for the quantitative valuation of Caspase 3-immunopositive PCs density (mm^2) in male; ns: not significant, *p<0.05, **p<0.01, ***p<0.001.

Experimental group		Experimental group	*p*-value
Control (0.66 ± 0.45)	*vs*	1st AKB-48 (5.00 ± 2.14)	ns
Control (0.66 ± 0.45)	*vs*	2nd AKB-48 (13.68 ± 2.02)	**
Control (0.66 ± 0.45)	*vs*	3rd AKB-48 (18.04 ± 2.34)	***
1st AKB-48 (5.00 ± 2.14)	*vs*	2nd AKB-48 (13.68 ± 2.02)	*
1st AKB-48 (5.00 ± 2.14)	*vs*	3rd AKB-48 (18.04 ± 2.34)	**
2nd AKB-48 (13.68 ± 2.02)	*vs*	3rd AKB-48 (18.04 ± 2.34)	ns

Table S12. Statistical analysis for the quantitative valuation of Caspase 3-immunopositive PCs density (mm^2) in female; ns: not significant, *p<0.05, **p<0.01, ***p<0.001.

Experimental group		Experimental group	*p*-value
Control (0.84 ± 0.62)	*vs*	1st AKB-48 (16.90 ± 3.57)	*
Control (0.84 ± 0.62)	*vs*	2nd AKB-48 (21.71 ± 3.93)	**
Control (0.84 ± 0.62)	*vs*	3rd AKB-48 (26.58 ± 3.17)	***
1st AKB-48 (16.90 ± 3.57)	*vs*	2nd AKB-48 (21.71 ± 3.93)	ns
1st AKB-48 (16.90 ± 3.57)	*vs*	3rd AKB-48 (26.58 ± 3.17)	ns
2nd AKB-48 (21.71 ± 3.93)	*vs*	3rd AKB-48 (26.58 ± 3.17)	ns

Table S13. Statistical analysis for the quantitative valuation of Caspase 3-immunopositive IGL cell density (mm^2) in male; ns: not significant, *p<0.05, ***p<0.001.

Experimental group		Experimental group	*p*-value
Control (16.74 ± 6.86)	*vs*	1st AKB-48 (152.82 ± 13.04)	***
Control (16.74 ± 6.86)	*vs*	2nd AKB-48 (163.49 ± 16.69)	***
Control (16.74 ± 6.86)	*vs*	3rd AKB-48 (218.40 ± 17.35)	***
1st AKB-48 (152.82 ± 13.04)	*vs*	2nd AKB-48 (163.49 ± 16.69)	ns
1st AKB-48 (152.82 ± 13.04)	*vs*	3rd AKB-48 (218.40 ± 17.35)	*
2nd AKB-48 (163.49 ± 16.69)	*vs*	3rd AKB-48 (218.40 ± 17.35)	ns

Table S14. Statistical analysis for the quantitative valuation of Caspase 3-immunopositive IGL cell density (mm^2) in female; **p<0.01, ***p<0.001.

Experimental group		Experimental group	*p*-value
Control (19.82 ± 3.39)	*vs*	1st AKB-48 (177.99 ± 6.19)	***
Control (19.82 ± 3.39)	*vs*	2nd AKB-48 (302.45 ± 24.19)	***
Control (19.82 ± 3.39)	*vs*	3rd AKB-48 (723.97 ± 37.30)	***
1st AKB-48 (177.99 ± 6.19)	*vs*	2nd AKB-48 (302.45 ± 24.19)	**
1st AKB-48 (177.99 ± 6.19)	*vs*	3rd AKB-48 (723.97 ± 37.30)	***
2nd AKB-48 (302.45 ± 24.19)	*vs*	3rd AKB-48 (723.97 ± 37.30)	***

Table S15. Statistical analysis for the quantitative valuation of BAX-immunopositive PCs cell density (mm^2) in male; ns: not significant, ***p<0.001.

Experimental group		Experimental group	*p*-value
Control (1.91 ± 0.39)	*vs*	1st AKB-48 (12.45 ± 2.58)	ns
Control (1.91 ± 0.39)	*vs*	2nd AKB-48 (42.83 ± 2.23)	***
Control (1.91 ± 0.39)	*vs*	3rd AKB-48 (67.65 ± 4.46)	***
1st AKB-48 (12.45 ± 2.58)	*vs*	2nd AKB-48 (42.83 ± 2.23)	***
1st AKB-48 (12.45 ± 2.58)	*vs*	3rd AKB-48 (67.65 ± 4.46)	***
2nd AKB-48 (42.83 ± 2.23)	*vs*	3rd AKB-48 (67.65 ± 4.46)	***

Table S16. Statistical analysis for the quantitative valuation of BAX-immunopositive PCs cell density (mm^2) in female; ns: not significant, *p<0.05, ***p<0.001.

Experimental group		Experimental group	*p*-value
Control (2.08 ± 0.45)	*vs*	1st AKB-48 (2.64 ± 0.79)	ns
Control (2.08 ± 0.45)	*vs*	2nd AKB-48 (12.05 ± 2.12)	*
Control (2.08 ± 0.45)	*vs*	3rd AKB-48 (44.52 ± 3.22)	***
1st AKB-48 (2.64 ± 0.79)	*vs*	2nd AKB-48 (12.05 ± 2.12)	*
1st AKB-48 (2.64 ± 0.79)	*vs*	3rd AKB-48 (44.52 ± 3.22)	***
2nd AKB-48 (12.05 ± 2.12)	*vs*	3rd AKB-48 (44.52 ± 3.22)	***

Table S17. Statistical analysis for the quantitative valuation of BAX-immunopositive PCs OD in male; ns: not significant, **p<0.01, ***p<0.001.

Experimental group		Experimental group	*p*-value
Control (24.13 ± 0.86)	*vs*	1st AKB-48 (26.54 ± 1.36)	ns
Control (24.13 ± 0.86)	*vs*	2nd AKB-48 (41.52 ± 0.76)	***
Control (24.13 ± 0.86)	*vs*	3rd AKB-48 (72.19 ± 2.60)	***
1st AKB-48 (26.54 ± 1.36)	*vs*	2nd AKB-48 (41.52 ± 0.76)	***
1st AKB-48 (26.54 ± 1.36)	*vs*	3rd AKB-48 (72.19 ± 2.60)	***
2nd AKB-48 (41.52 ± 0.76)	*vs*	3rd AKB-48 (72.19 ± 2.60)	**

Table S18. Statistical analysis for the quantitative valuation of BAX-immunopositive PCs OD in female; ns: not significant, ***p<0.001.

Experimental group		Experimental group	*p*-value
Control (26.75 ± 1.25)	*vs*	1st AKB-48 (28.65 ± 1.77)	ns
Control (26.75 ± 1.25)	*vs*	2nd AKB-48 (43.97 ± 1.41)	ns
Control (26.75 ± 1.25)	*vs*	3rd AKB-48 (62.55 ± 3.51)	***
1st AKB-48 (28.65 ± 1.77)	*vs*	2nd AKB-48 (43.97 ± 1.41)	***
1st AKB-48 (28.65 ± 1.77)	*vs*	3rd AKB-48 (62.55 ± 3.51)	***
2nd AKB-48 (43.97 ± 1.41)	*vs*	3rd AKB-48 (62.55 ± 3.51)	ns

Table S19. Statistical analysis for the quantitative valuation of Bcl-2-immunopositive ML OD in male; *p<0.05, ***p<0.001.

Experimental group		Experimental group	*p*-value
Control (52.35 ± 3.95)	*vs*	1st AKB-48 (27.39 ± 2.09)	*
Control (52.35 ± 3.95)	*vs*	2nd AKB-48 (14.15 ± 1.28)	***
Control (52.35 ± 3.95)	*vs*	3rd AKB-48 (5.71 ± 0.54)	***
1st AKB-48 (27.39 ± 2.09)	*vs*	2nd AKB-48 (14.15 ± 1.28)	*
1st AKB-48 (27.39 ± 2.09)	*vs*	3rd AKB-48 (5.71 ± 0.54)	***
2nd AKB-48 (14.15 ± 1.28)	*vs*	3rd AKB-48 (5.71 ± 0.54)	*

Table S20. Statistical analysis for the quantitative valuation of Bcl-2-immunopositive ML OD in female; ns: not significant, **p<0.01, ***p<0.001.

Experimental group		Experimental group	*p*-value
Control (38.06 ± 0.96)	*vs*	1st AKB-48 (22.05 ± 1.27)	***
Control (38.06 ± 0.96)	*vs*	2nd AKB-48 (16.38 ± 1.29)	***
Control (38.06 ± 0.96)	*vs*	3rd AKB-48 (6.83 ± 0.97)	***
1st AKB-48 (22.05 ± 1.27)	*vs*	2nd AKB-48 (16.38 ± 1.29)	ns
1st AKB-48 (22.05 ± 1.27)	*vs*	3rd AKB-48 (6.83 ± 0.97)	***
2nd AKB-48 (16.38 ± 1.29)	*vs*	3rd AKB-48 (6.83 ± 0.97)	**

Table S21. Statistical analysis for the quantitative valuation of Bcl-2-immunopositive mossy fiber rosettes OD in male; **p<0.01, ***p<0.001.

Experimental group		Experimental group	*p*-value
Control (37.02 ± 0.66)	*vs*	1st AKB-48 (31.82 ± 1.30)	**
Control (37.02 ± 0.66)	*vs*	2nd AKB-48 (22.76 ± 1.44)	***
Control (37.02 ± 0.66)	*vs*	3rd AKB-48 (14.73 ± 0.58)	***
1st AKB-48 (31.82 ± 1.30)	*vs*	2nd AKB-48 (22.76 ± 1.44)	***
1st AKB-48 (31.82 ± 1.30)	*vs*	3rd AKB-48 (14.73 ± 0.58)	***
2nd AKB-48 (22.76 ± 1.44)	*vs*	3rd AKB-48 (14.73 ± 0.58)	***

Table S22. Statistical analysis for the quantitative valuation of Bcl-2-immunopositive mossy fiber rosettes OD in female; ns: not significant, *p<0.05, ***p<0.001.

Experimental group		Experimental group	*p*-value
Control (36.20 ± 1.72)	*vs*	1st AKB-48 (33.83 ± 1.83)	ns
Control (36.20 ± 1.72)	*vs*	2nd AKB-48 (21.11 ± 1.44)	***
Control (36.20 ± 1.72)	*vs*	3rd AKB-48 (15.06 ± 0.62)	***
1st AKB-48 (33.83 ± 1.83)	*vs*	2nd AKB-48 (21.11 ± 1.44)	***
1st AKB-48 (33.83 ± 1.83)	*vs*	3rd AKB-48 (15.06 ± 0.62)	***
2nd AKB-48 (21.11 ± 1.44)	*vs*	3rd AKB-48 (15.06 ± 0.62)	*

Table S23. Statistical analysis for the quantitative valuation of AIF-immunoposite ML OD in male; ns: not significant, ***p<0.001.

Experimental group		Experimental group	*p*-value
Control (17.09 ± 0.65)	*vs*	1st AKB-48 (16.91 ± 0.58)	ns
Control (17.09 ± 0.65)	*vs*	2nd AKB-48 (29.50 ± 0.78)	***
Control (17.09 ± 0.65)	*vs*	3rd AKB-48 (30.01 ± 0.80)	***
1st AKB-48 (16.91 ± 0.58)	*vs*	2nd AKB-48 (29.50 ± 0.78)	***
1st AKB-48 (16.91 ± 0.58)	*vs*	3rd AKB-48 (30.01 ± 0.80)	***
2nd AKB-48 (29.50 ± 0.78)	*vs*	3rd AKB-48 (30.01 ± 0.80)	ns

Table S24. Statistical analysis for the quantitative valuation of AIF-immunoposite ML OD in female; ns: not significant, ***p<0.001.

Experimental group		Experimental group	*p*-value
Control (17,57 ± 0,75)	*vs*	1st AKB-48 (17,82 ± 0,50)	ns
Control (17,57 ± 0,75)	*vs*	2nd AKB-48 (30,09 ± 2,00)	***
Control (17,57 ± 0,75)	*vs*	3rd AKB-48 (29,88 ± 1,23)	***
1st AKB-48 (17,82 ± 0,50)	*vs*	2nd AKB-48 (30,09 ± 2,00)	***
1st AKB-48 (17,82 ± 0,50)	*vs*	3rd AKB-48 (29,88 ± 1,23)	***
2nd AKB-48 (30,09 ± 2,00)	*vs*	3rd AKB-48 (29,88 ± 1,23)	ns

Table S25. Statistical analysis for the quantitative valuation of AIF-immunopositive PCs cell density (mm^2) in male; ns: not significant, ***p<0.001.

Experimental group		Experimental group	*p*-value
Control (3.32 ± 1.77)	*vs*	1st AKB-48 (4.56 ± 1.54)	ns
Control (3.32 ± 1.77)	*vs*	2nd AKB-48 (9.45 ± 2.37)	ns
Control (3.32 ± 1.77)	*vs*	3rd AKB-48 (59.63 ± 3.36)	***
1st AKB-48 (4.56 ± 1.54)	*vs*	2nd AKB-48 (9.45 ± 2.37)	ns
1st AKB-48 (4.56 ± 1.54)	*vs*	3rd AKB-48 (59.63 ± 3.36)	***
2nd AKB-48 (9.45 ± 2.37)	*vs*	3rd AKB-48 (59.63 ± 3.36)	***

Table S26. Statistical analysis for the quantitative valuation of AIF-immunopositive PCs cell density (mm^2) in female; ns: not significant, ***p<0.001.

Experimental group		Experimental group	*p*-value
Control (2.89 ± 1.58)	*vs*	1st AKB-48 (4.92 ± 1.44)	ns
Control (2.89 ± 1.58)	*vs*	2nd AKB-48 (7.85 ± 1.29)	ns
Control (2.89 ± 1.58)	*vs*	3rd AKB-48 (60.97 ± 1.28)	***
1st AKB-48 (4.92 ± 1.44)	*vs*	2nd AKB-48 (7.85 ± 1.29)	ns
1st AKB-48 (4.92 ± 1.44)	*vs*	3rd AKB-48 (60.97 ± 1.28)	***
2nd AKB-48 (7.85 ± 1.29)	*vs*	3rd AKB-48 (60.97 ± 1.28)	***

Table S27. Statistical analysis for the quantitative valuation of AIF-immunopositive PCs OD in male; ns: not significant, ***p<0.001.

Experimental group		Experimental group	*p*-value
Control (34.41 ± 1.30)	*vs*	1st AKB-48 (35.75 ± 0.93)	ns
Control (34.41 ± 1.30)	*vs*	2nd AKB-48 (34.45 ± 1.23)	ns
Control (34.41 ± 1.30)	*vs*	3rd AKB-48 (63.47 ± 3.05)	***
1st AKB-48 (35.75 ± 0.93)	*vs*	2nd AKB-48 (34.45 ± 1.23)	ns
1st AKB-48 (35.75 ± 0.93)	*vs*	3rd AKB-48 (63.47 ± 3.05)	***
2nd AKB-48 (34.45 ± 1.23)	*vs*	3rd AKB-48 (63.47 ± 3.05)	***

Table S28. Statistical analysis for the quantitative valuation of AIF-immunopositive PCs OD in female; ns: not significant, ***p<0.001.

Experimental group		Experimental group	*p*-value
Control (34,25 ± 0,93)	*vs*	1st AKB-48 (33,71 ± 1,74)	ns
Control (34,25 ± 0,93)	*vs*	2nd AKB-48 (34,93 ± 1,49)	ns
Control (34,25 ± 0,93)	*vs*	3rd AKB-48 (63,24 ± 1,81)	***
**1st AKB-48 (33,71 ± 1,74)**	*vs*	2nd AKB-48 (34,93 ± 1,49)	ns
**1st AKB-48 (33,71 ± 1,74)**	*vs*	3rd AKB-48 (63,24 ± 1,81)	***
**2nd AKB-48 (34,93 ± 1,49)**	*vs*	3rd AKB-48 (63,24 ± 1,81)	***

Table S29. Statistical analysis for the quantitative valuation of OPA1-immunopositive PCs cell density (mm^2) in male; ns: not significant, *p<0.05, **p<0.01, ***p<0.001.

Experimental group		Experimental group	*p*-value
Control (58.78 ± 2.10)	*vs*	1st AKB-48 (54.57 ± 2.00)	ns
Control (58.78 ± 2.10)	*vs*	2nd AKB-48 (45.06 ± 1.85)	***
Control (58.78 ± 2.10)	*vs*	3rd AKB-48 (32.50 ± 1.33)	***
1st AKB-48 (54.57 ± 2.00)	*vs*	2nd AKB-48 (45.06 ± 1.85)	*
1st AKB-48 (54.57 ± 2.00)	*vs*	3rd AKB-48 (32.50 ± 1.33)	***
2nd AKB-48 (45.06 ± 1.85)	*vs*	3rd AKB-48 (32.50 ± 1.33)	**

Table S30. Statistical analysis for the quantitative valuation of OPA1-immunopositive PCs cell density (mm^2) in female; ns: not significant, **p<0.01, ***p<0.001.

Experimental group		Experimental group	*p*-value
Control (59.66 ± 1.28)	*vs*	1st AKB-48 (52.89 ± 3.04)	ns
Control (59.66 ± 1.28)	*vs*	2nd AKB-48 (46.55 ± 1.85)	**
Control (59.66 ± 1.28)	*vs*	3rd AKB-48 (30.59 ± 1.26)	***
1st AKB-48 (52.89 ± 3.04)	*vs*	2nd AKB-48 (46.55 ± 1.85)	ns
1st AKB-48 (52.89 ± 3.04)	*vs*	3rd AKB-48 (30.59 ± 1.26)	***
2nd AKB-48 (46.55 ± 1.85)	*vs*	3rd AKB-48 (30.59 ± 1.26)	***

Table S31. Statistical analysis for the quantitative valuation of OPA1-immunopositive PCs OD in male; *p<0.05, ***p<0.001.

Experimental group		Experimental group	*p*-value
Control (118.32 ± 4.58)	*vs*	1st AKB-48 (102.83 ± 4.83)	*
Control (118.32 ± 4.58)	*vs*	2nd AKB-48 (71.13 ± 2.52)	***
Control (118.32 ± 4.58)	*vs*	3rd AKB-48 (50.25 ± 1.94)	***
1st AKB-48 (102.83 ± 4.83)	*vs*	2nd AKB-48 (71.13 ± 2.52)	***
1st AKB-48 (102.83 ± 4.83)	*vs*	3rd AKB-48 (50.25 ± 1.94)	***
2nd AKB-48 (71.13 ± 2.52)	*vs*	3rd AKB-48 (50.25 ± 1.94)	***

Table S32. Statistical analysis for the quantitative valuation of OPA1-immunopositive PCs OD in female; ns: not significant, ***p<0.001.

Experimental group		Experimental group	*p*-value
Control (120.16 ± 4.56)	*vs*	1st AKB-48 (106.56 ± 5.30)	ns
Control (120.16 ± 4.56)	*vs*	2nd AKB-48 (65.64 ± 3.04)	***
Control (120.16 ± 4.56)	*vs*	3rd AKB-48 (51.18 ± 2.78)	***
1st AKB-48 (106.56 ± 5.30)	*vs*	2nd AKB-48 (65.64 ± 3.04)	***
1st AKB-48 (106.56 ± 5.30)	*vs*	3rd AKB-48 (51.18 ± 2.78)	***
2nd AKB-48 (65.64 ± 3.04)	*vs*	3rd AKB-48 (51.18 ± 2.78)	ns
